# Mycological Profile and Associated Factors Among Patients with Dermatophytosis in Astana, Kazakhstan

**DOI:** 10.3390/jof11010065

**Published:** 2025-01-16

**Authors:** Alma Aimoldina, Ainura Smagulova, Gulnar Batpenova, Nellie Konnikov, Togzhan Algazina, Zulfiya Jetpisbayeva, Dinara Azanbayeva, Darkhan Amantayev, Vladimir Kiyan

**Affiliations:** 1 Department of Dermatovenereology and Dermatocosmetology, Astana Medical University, Astana 010011, Kazakhstan; aimoldina.a@amu.kz (A.A.); batpenova.g@amu.kz (G.B.); khabdina.t@amu.kz (T.A.); jetpisbaeva.z@amu.kz (Z.J.); azanbayeva.d@amu.kz (D.A.); amantayev.d@amu.kz (D.A.); 2 Laboratory of Biodiversity and Genetic Resources, National Center for Biotechnology, Astana 010011, Kazakhstan; smagulova0114@gmail.com; 3 Dermatology Section, Department of Medicine, VA Boston Healthcare System, Boston, MA 02130, USA; nellie.konnikov@va.gov; 4 Department of Dermatology, Tufts University School of Medicine, Boston, MA 02111, USA

**Keywords:** dermatophytosis, tinea, dermatophytes, mycological profile, risk factors, Kazakhstan

## Abstract

Dermatophytosis, also known as Tinea infection, remains a significant interdisciplinary concern worldwide. This dermatophyte infection may be more serious in individuals with underlying somatic diseases, immunodeficiencies, endocrine disorders, or chronic illnesses. This study analyzed 313 patients with suspected dermatophytosis. Data were gathered through questionnaires and medical records were reviewed. Biological samples were cultured on Sabouraud dextrose agar, and PCR was employed to assess the genetic diversity of strains. Statistical analysis was conducted using SPSS version 26. The overall prevalence of dermatophytosis in the cohort was 30.4%. Among the cultured isolates, 73.7% were identified as *Microsporum canis*, while 26.3% were identified as *Trichophyton* species, including *T. mentagrophytes*, *T. tonsurans*, and *T. verrucosum*. Several factors were significantly associated with an increased risk of dermatophytosis, including the following: male gender (AOR = 1.97), age 1–10 years (AOR = 3.80), living in rural areas (AOR = 2.30), visiting public bathhouses (AOR = 2.32), visiting massage parlors (AOR = 1.39), contact with cats (AOR = 2.32), family history of dermatophytosis (AOR = 3.04), and sexual contact with an infected or unknown partner (AOR = 3.08). Dermatophytosis was identified in approximately one third of the patients by culture (30.4%), with the risk heightened in individuals under 10 years old (43.6%), those living in rural areas (33.3%), and those with a family history of dermatophytosis (35.7%) or close contact with cats (39.4%). The findings underscore the need for strengthened preventive measures and targeted diagnostics, particularly among high-risk groups.

## 1. Introduction

Dermatophytosis, commonly referred to as Tinea infection, is a significant interdisciplinary health issue worldwide. Its prevalence is particularly high in tropical regions, where factors such as high humidity, overcrowding, and poor hygiene contribute to its spread [1]. According to the World Health Organization, one in three people globally suffers from mycosis, and 90% of individuals have experienced a fungal infection at least once in their lifetime. Among superficial mycoses, lesions of the skin, nails, and hair are the most common. Developing countries, such as India, are experiencing an increase in dermatophyte infections, along with the growing challenge of managing chronic and recurrent cases [2]. Currently, superficial mycoses of the skin affect approximately 20% of the global population [3]. For many countries, fungal infections caused by *Trichophyton* and *Microsporum* species represent a significant public health challenge [4,5,6]. In Kazakhstan, the issue is similarly pressing. Data from the S. Kairbekova National Scientific Center for Health Development reported an incidence rate of 63.3 cases of dermatomycosis per 100,000 people in 2023 [7].

The transmissibility of dermatophytosis pathogens varies across the different forms of the infection. Mycosis of the scalp is the most contagious, whereas dermatophytoses of the trunk, hands, and groin are significantly less transmissible [1]. A growing global public health concern is the rise of treatment-resistant dermatophytoses, particularly those caused by *Trichophyton rubrum* and *Trichophyton mentagrophytes*. This issue is notably prevalent in endemic regions such as India [8], with cases also emerging in Europe and other developed countries [9,10]. Research indicates that the resistance of fungal pathogens to antifungal drugs is primarily genetic in origin, which often contributes to the ineffectiveness of antifungal therapies in treating dermatomycosis [11]. Furthermore, there is increasing evidence of new fungal pathogens and atypical forms of dermatophytosis, along with a rise in the number of cases resistant to standard treatments [12].

Infection can be transmitted by infected individuals or through contact with animals, including pets, cattle, and small livestock [1,13]. Geographic location, healthcare, immigration, climate (temperature, humidity, wind, etc.), overcrowding, environmental hygiene culture, awareness to dermatophytes, the age of individuals, hygiene, and socioeconomic conditions have been described as major factors for these variations in dermatophyte epidemiology [4]. The clinical presentation of mycosis is influenced by several factors, including the type and pathogenicity of the causative organism, the immune status of the host, and the appropriateness of the therapy. Without proper treatment, the disease may progress to a chronic form [14].

Given the rising incidence of dermatomycosis and the increasing prevalence of resistant and persistent forms of dermatophyte infections, we conducted a study to evaluate the mycological profile and associated factors among patients with dermatophytosis in Astana, Kazakhstan. Assessing morbidity in relation to patients’ gender, age, and other demographic factors will enhance the development of sanitary, anti-epidemic, and preventive strategies aimed at reducing the prevalence of contagious dermatoses in the population.

## 2. Materials and Methods

### 2.1. Research Area and Period

This study was conducted in the dermatology unit of Astana City Multidisciplinary Hospital #3 between February and December 2023. Ethical approval for this study involving human participants was obtained from the local ethics committee of Astana Medical University (decision #5, meeting #2, dated 23 February 2023). Written informed consent was voluntarily obtained from each participant or their legal guardian after a detailed explanation of the study’s purpose. Participants were assured of confidentiality, and anonymity was maintained throughout the study by ensuring that completed questionnaires remained unidentified.

### 2.2. Study Design and Populations

This study included 313 patients of both sexes and all age groups with suspected dermatophytosis who visited the dermatology unit of City Multidisciplinary Hospital No. 3 in Astana, Kazakhstan, between January and December 2023. The study population consisted of children aged 2 to 18 years, men aged 19 to 75 years, and women aged 19 to 83 years. Data collection encompassed medical history, life history, epidemiological information, and objective clinical examinations of the patients. Patients who had previously received treatment for fungal infections or whose medical records were incomplete were excluded from the study.

### 2.3. Data Collection Methods

Data were collected using a pre-tested, structured questionnaire developed based on a comprehensive review of the relevant literature. Written informed consent was obtained from the participants or their guardians prior to data collection, which was conducted simultaneously with the collection of biological samples. Additionally, patients’ medical records were reviewed to gather further insights into social and epidemiological factors.

### 2.4. Collection and Transportation of Samples

Skin scrapings, including epidermal scales and infected hairs, were collected by laboratory researchers using a sterile scalpel blade. The lesions were first treated with 70% ethyl alcohol before scraping. Each sample was labeled and transported in paper packets for culture within 24 h.

### 2.5. Microbiological Research

Samples of biological material (hair and scales) were collected from the affected area of the skin on day 0 by scraping and were delivered to the mycological laboratory. The samples were sown on a nutrient medium (Sabouraud culture) with dextrose agar. During the initial isolation of the pathogen (1 day) and upon the receipt of a pure culture, surface cultivation of the fungus was carried out at a temperature of 28 °C for at least 6 days until the formation of characteristic colonies. Mycological diagnosis was made 10 to 20 days later. The types of pathogens were determined based on the cultural and morphological characteristics of the colonies, as well as microscopic morphology.

### 2.6. Genetic Identification

Species identification was conducted exclusively on the isolated strains. Polymerase chain reaction (PCR) was performed to identify the genetic diversity of all isolated dermatophytes using 1 primer pair targeting ribosomal RNA genes: forward *ITS1* (5’-TCCGTAGGTGAACCTGCGG-3’) and reverse *ITS4* (5’-TCCTCCGCTTATTGATATGC-3’) [15]. For the precise identification of *Trichophyton* dermatophyte species, gene sequences were utilized as probes to sequence the *ITS* region of the rDNA: V9G 5’-TTACGTCCCTGCCCTTTGTA-3’ and LSU266 5’-GCATTCCCAAACAACTCGACTC-3’ [16]. The PCR-amplified target gene fragment was purified using a Quick PCR Purification Kit (Invitrogen, Vilnius, Lithuania) following the manufacturer’s protocol. Sequencing was performed using a Seq Studio Genetic Analyzer (Thermo Fisher Scientific Applied Biosystems, Marsiling Industrial Estate Road 3, #07-06, Singapore) according to the manufacturer’s instructions. The resulting nucleotide sequences were visually checked using BioCapt software (version 11.0). The sequences were deposited in GenBank with accession nos. PQ844756, PQ844807, PQ844808, PQ844810, PQ844811, PQ844827, PQ844828, and PQ844831–PQ844834 (*M. canis*); PQ844693, PQ844702, PQ849825 (*T. mentagrophytes*), PQ844689, PQ844692, PQ844695, and PQ844699 (*T. tonsurans*); and PQ844679 and PQ844696 (*T. verrucosum*). The nucleotide sequences of the studied species were compared with other sequences in the NCBI GenBank database using BLAST analysis: *M. canis* (EF631606), *T. mentagrophytes* (MT561384, MZ614625), *T. tonsurans* (AF170476), and *T. verrucosum* (OW984892).

## 3. Results

### 3.1. Social and Demographic Characteristics

A total of 313 patients with a suspected diagnosis of dermatophytosis were included in this study. Suspected dermatophytosis refers to a clinical condition where dermatophytosis is considered a possible diagnosis based on clinical signs and symptoms. Confirmed dermatophytosis refers to a condition where the presence of dermatophytes has been verified through laboratory confirmation, most commonly using culture testing. The participants’ ages ranged from 2 to 83 years, with an average age of 23.0 (±16.8) years. Among the patients, 175 (55.9%) were men and 138 (44.1%) were women. The majority of the participants were schoolchildren (104 individuals, 33.2%), followed by office workers (63 individuals, 20.1%). Additionally, 42 children (13.4%) attended kindergarten. The vast majority of the patients resided in the city (256 individuals, 81.8%) (Table 1).

### 3.2. Clinical Characteristics

We evaluated the prevailing behavior patterns and contact situations among the study participants over the past month. According to our survey, 42 participants (13.4%) reported household contact with a relative diagnosed with dermatophytosis, while 48 participants (15.3%) reported sexual contact with an infected or unknown partner. Among the 313 participants, 94 individuals (30.0%) had contact with cats (either stray or domestic), and 45 individuals (14.4%) reported contact with farm animals, including sheep and cows. Additionally, 59 participants (18.8%) were involved in sports activities, such as judo or freestyle wrestling. Notably, 56 participants (17.9%) reported visiting baths, and 34 participants (10.9%) frequented massage parlors (Table 2).

### 3.3. Extent of Dermatophytosis

**Figure 1:**
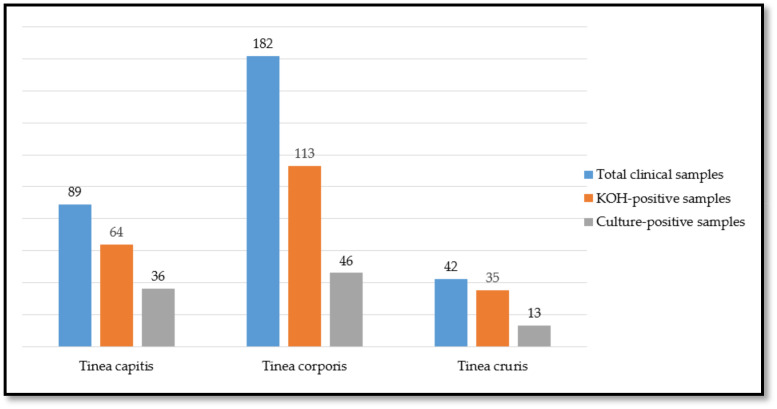
The relationship between the initial clinical suspicion and the laboratory results.

Among the 313 cases of dermatophytosis, fungi were detected in 67.7% of cases through direct KOH microscopy and in 30.4% through culture. Based on these findings, 30.4% of the suspected cases were confirmed as dermatophytosis through culture testing (Figure 1).

**Figure 2:**
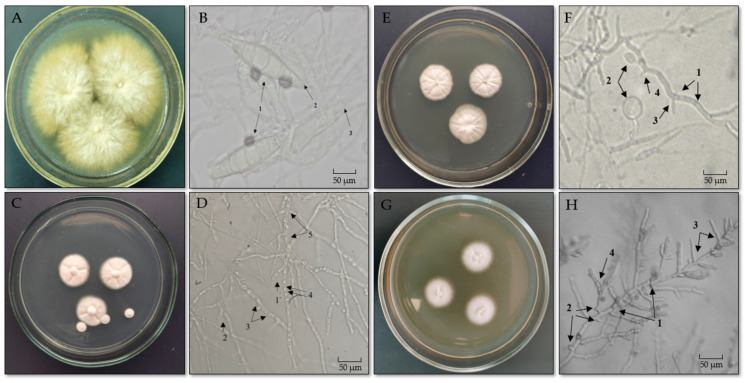
Growth of pathogenic fungi on Sabouraud dextrose agar and their microscopic structures: (**A**)—*M. canis* colonies on Sabouraud agar. (**B**)—Microscopic structures of *M. canis*: 1—spindle-shaped macroconidia; 2—conidiophore; 3—septate mycelium. (**C**)—*T. mentagrophytes* colonies on Sabouraud agar. (**D**)—Microscopic structures of *T. mentagrophytes*: 1—macroconidium; 2—microconidium; 3—septate mycelium; 4—formation of arthrospores; 5—formation of chlamydospores. (**E**)—*T. verrucosum* colonies on Sabouraud agar. (**F**)—Microscopic structures of *T. verrucosum*: 1—septate mycelium; 2—chlamydospores; 3—macroconidium; 4—microconidium. (**G**)—*T. tonsurans* colonies on Sabouraud agar. (**H**)—Microscopic structures of *T. tonsurans*: 1—septate mycelium; 2—microconidia; 3—macroconidia; 4—formation of arthrospores.

Of the cultured fungal isolates, 73.7% were identified as *Microsporum canis*, and others were *Trichophyton* spp.—*T. mentagrophytes*, *T. tonsurans*, and *T. verrucosum* (Figure 2).

Tinea corporis was represented by the species *M. canis*, *T. mentagrophytes*, *T. tonsurans*, and *T. verrucosum*; Tinea capitis was represented by the species *M. canis*, *T. verrucosum*, and *T. tonsurans*; and Tinea cruris was represented solely by *T. mentagrophytes*.

A nucleotide BLAST analysis of *M. canis* strain 48’s sequences revealed a maximum homology of 99–100% with *M. canis* sequences from China (EF631606) and Cambodia (MT790277). *T. mentagrophytes* strain 273’s sequences exhibited a maximum homology of 98% with *T. mentagrophytes* sequences from India (MT561384), *T. mentagrophytes* strain 157’s sequences showed 99% homology and *T. mentagrophytes* strain 194’s sequences showed 94% homology with sequences from Germany (MZ614625). *T. tonsurans* strain 52’s sequences showed a maximum homology of 99–100% with *T. tonsurans* sequences from Canada (AF170476) and Brazil (MN295945). Finally, *T. verrucosum* strain 009’s sequences demonstrated a maximum homology of 99–100% with *T. verrucosum* sequences from Belgium (OW984892) and Egypt (MT260175).

**Figure 3:**
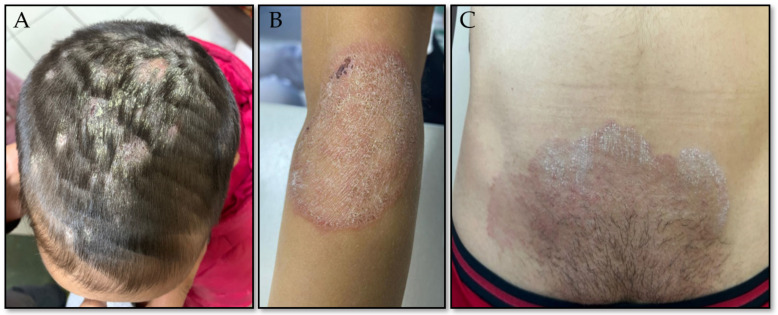
Clinical presentation of dermatophytosis affecting different body regions: (**A**)—scalp; (**B**)—trunk; (**C**)—groin area.

The culture results indicated the highest prevalence among males (70.53%), the 1–10-year age group (43.6%), rural residents (33.3%), and schoolchildren (40.4%). Tinea corporis was the most common clinical manifestation, occurring in 45.1% of males and 67.1% of females (Table 3). Figure 3 depicts clinical photographs illustrating dermatophytosis of the scalp, trunk, and groin area, respectively.

### 3.4. Factors Associated with Dermatophytosis

Gender, age, place of residence, occupation, contact with a sick relative, contact with a cat, sexual contact with an infected or unknown partner, contact with farm animals, visiting a sauna, visiting a massage parlor, and participating in sports clubs were identified as potential predictors of dermatophytosis in the bivariate regression analysis (*p* < 0.25). These variables were included in the multivariate logistic regression analysis. In the multivariate analysis, gender, age, place of residence, contact with a sick relative, contact with a cat, sexual contact with an infected or unknown partner, visiting a sauna, visiting a massage parlor, and participation in sports clubs remained statistically significant (*p* < 0.05).

Men were nearly twice as likely to develop dermatophytosis compared to women (AOR = 1.97; 95% CI: 1.14–3.39). Children aged 1–10 years were at a significantly higher risk, being 3.8 times more likely to be infected than individuals aged over 30 years (AOR = 3.80; 95% CI: 1.01–4.31). Rural residents were 2.3 times more likely to develop dermatophytosis compared to urban residents (AOR = 2.30; 95% CI: 1.04–5.09).

Behavioral factors also played a crucial role. Visiting saunas and massage parlors increased the risk of dermatophytosis by 2.32 times (AOR = 2.32; 95% CI: 1.02–5.28) and 1.39 times (AOR = 1.39; 95% CI: 1.23–8.28), respectively. Contact with cats raised the likelihood of infection by 2.32 times (AOR = 2.32; 95% CI: 1.12–4.81), while household contact with a relative with dermatophytosis increased the risk by 3.04 times (AOR = 3.04; 95% CI: 1.22–7.55). Sexual contact with an infected or unknown partner posed a similarly elevated risk (AOR = 3.08; 95% CI: 1.34–7.10). Participants attending sports clubs, such as those practicing freestyle wrestling or judo, were 3.21 times more likely to develop the disease than those not participating in such activities (AOR = 3.21; 95% CI: 1.26–8.14).

In contrast, factors such as occupation and contact with farm animals did not show a statistically significant association with dermatophytosis in this study (Table 4).

## 4. Discussion

A clinical and mycological study was conducted on 313 patients with suspected dermatophytosis in the dermatology unit of City Multidisciplinary Hospital No. 3, Astana, Kazakhstan. Dermatophytes were detected in 67.7% of cases using direct KOH microscopy and in 30.4% by culture. These findings underscore the importance of both diagnostic methods; while direct microscopy non-specifically detects all fungi, culture provides definitive species identification.

In this study, the prevalence of dermatophytosis confirmed by culture was 30.4%, comparable to findings from Karachi, Pakistan (31.29%) [17], and the Tertiary Care Teaching Institute in India (32.0%) [18]. Higher prevalence rates have been reported in Addis Ababa, Ethiopia (46.5%) [4], and Gujarat, India (83.33%) [19], potentially reflecting differences in sample size, study populations, diagnostic methods, and health awareness levels among participants.

The discrepancy between the clinical and laboratory diagnoses of dermatophytosis is influenced by several factors. Clinically, dermatophytosis is characterized by symptoms such as erythema, scaling, and pruritus, which are suggestive of fungal infection. However, definitive diagnosis necessitates laboratory confirmation, commonly through direct microscopic examination or culture. Instances of negative culture results despite positive findings on direct microscopic examination may be attributed to insufficient fungal load, wherein the quantity of fungal material in the sample is inadequate for successful culture growth. Furthermore, prior self-medication can significantly reduce the fungal burden, resulting in culture negativity despite the presence of fungal structures visible under the microscope. Addressing such diagnostic challenges requires a systematic approach. When direct microscopic findings are positive but culture results are negative, clinicians should consider repeat sampling and ongoing patient monitoring. Incorporating advanced diagnostic techniques, such as PCR or fungal antigen detection assays, may improve diagnostic accuracy. Additionally, a thorough patient history, particularly regarding prior antifungal use, is crucial for interpreting laboratory results and optimizing clinical management.

The genus *Microsporum* was the predominant isolate in this study, accounting for 73.7% of cases, followed by *Trichophyton* at 26.3%. This aligns with findings from other global studies [11,20], though a predominance of *Trichophyton* was reported elsewhere [21,22]. For instance, in Ethiopia, *Trichophyton* spp. accounted for 79.2% of isolates, with *Microsporum* and *Epidermophyton* species comprising the rest [23]. These variations may be influenced by regional prevalence, climatic and environmental factors, and diagnostic techniques.

In the present study, men were observed to have a higher risk of developing dermatophyte infections. This aligns with findings from studies conducted in Korea [24] and Ethiopia [23]. This increased risk among men may stem from physiological factors such as oilier skin and increased sweating, as well as behavioral factors like engagement in active sports, wearing tight clothing, and paying less attention to personal hygiene compared to women. However, our findings differ from those in Salem, India [25], and Karachi, Pakistan [26], where women had a higher prevalence of dermatophytosis. Additionally, men’s tendency to delay seeking medical attention for skin conditions could exacerbate the infection’s spread and severity.

Children under 10 years of age in our study exhibited a higher rate of dermatophyte infection. Similar observations have been reported in studies from Pakistan [16], the UK [27], and India [28]. However, other research has highlighted an increased risk in older age groups, such as 11–20 years [29] and 21–30 years [30]. This higher susceptibility in young children could be attributed to an underdeveloped immune system, increased physical activity, frequent environmental contact, and inadequate hygiene practices, which collectively create favorable conditions for fungal infections.

Our study also found a higher prevalence of dermatophytosis in rural populations compared to urban areas, consistent with findings from northern India [31] and Sudan [29]. Contributing factors may include regular contact with animals, high humidity, outdoor work leading to skin injuries, and limited healthcare access in rural settings. Poor hygiene and exposure to damp, unsanitary working conditions are significant risk factors, especially for chronic or untreated cases. Contrastingly, a study from southwestern Nigeria [32] reported higher infection rates in urban residents, possibly reflecting regional differences in healthcare access and environmental conditions.

In this study, household contact with a relative diagnosed with dermatophytosis emerged as a significant risk factor for the disease, consistent with findings from studies in India [33,34,35]. This increased risk can be attributed to the frequent close interactions within families, which facilitate the spread of infection. Additionally, the shared use of household items such as towels, combs, pillows, and hats further elevates the likelihood of transmission among relatives. Our data also revealed that patients with a history of contact with cats had a 2.32-fold higher risk of developing dermatophytosis compared to those without such contact. This aligns with findings from studies in Israel [36], Algeria [37], and Poland [38]. Cats are known to be asymptomatic carriers of dermatophytes, particularly when conditions such as poor hygiene, weakened immunity, or crowding in breeding farms or shelters exist. Transmission occurs through direct contact with infected animals or indirectly via contaminated objects in the environment. Children are especially vulnerable due to frequent close interactions with pets and less stringent hygiene practices [39].

Sexual contact with an infected or unknown partner was also found to significantly increase the risk of dermatophytosis. This is corroborated by studies from Germany [40], the USA [41], and Austria [42], which highlighted the potential for transmission through infected skin and mucous membranes during sexual activity. Our study determined that the likelihood of developing dermatophytosis through sexual contact with an infected partner increased by 3.08 times. While this emphasizes a noteworthy association, it is important to note that dermatophytosis primarily spreads through contact with contaminated surfaces, such as towels, footwear, and communal areas like showers and swimming pools [43].

However, our findings exemplify the critical importance of accurate pathogen identification for effective treatment, especially when caused by *Trichophyton indotineae*. *T. indotineae* has emerged as a significant public health concern due to its increasing resistance to antifungal agents and its ability to mimic the clinical presentation of sexually transmitted infections, particularly in the genital region. Misdiagnosis in such cases can lead to delayed treatment, the inappropriate use of antibiotics or other non-antifungal therapies, and the potential worsening of the infection [44]. In our study, we identified *T. mentagrophytes* strain 273, which exhibited a maximum homology of 98% with *T. mentagrophytes* sequences from India (Tinea cruris, MT561384) and 95% with *T. mentagrophytes* type VIII from Iraq (Tinea cruris, MT367568). This suggests that additional research is needed to study the circulation of the *T. indotineae* pathogen in our country. Given these challenges, it is essential to prioritize laboratory confirmation to accurately identify the causative agent. Advanced diagnostic methods, such as PCR and antifungal susceptibility testing, can be particularly valuable in detecting resistant strains and guiding effective therapy. Moreover, clinicians should remain vigilant when assessing infections in the genital region and consider dermatophytosis in their differential diagnosis to ensure timely and targeted treatment.

Lastly, visiting massage parlors was identified as a potential risk factor for dermatophytosis. This finding aligns with global research, which identifies spas and similar establishments as environments conducive to the spread of dermatophytosis [45,46,47]. High humidity levels, prolonged exposure to potentially contaminated surfaces, and close contact with individuals in such settings create favorable conditions for fungal transmission.

This study identified an increased risk of dermatophytosis among individuals participating in sports, particularly contact sports such as wrestling and judo. This heightened risk is likely associated with close physical contact, frequent mechanical injuries, and the suboptimal hygiene or maintenance in sports facilities, which collectively foster an environment conducive to fungal transmission. Similar findings have been reported in a study from China documenting an outbreak of Tinea capitis among wrestlers [48] and in a meta-analysis by Kermani et al. focusing on *Tinea gladiatorum* [49]. These studies underscore the necessity of maintaining stringent hygiene protocols in sports facilities and providing timely treatment for fungal infections to prevent their propagation and associated complications.

Visiting bathhouses also emerged as a significant risk factor for dermatophytosis in our study. Facilities such as hammams, swimming pools, and saunas inherently provide an ideal environment for fungal growth given their high humidity, elevated temperatures, and potential lapses in hygiene. Studies have identified pathogenic fungi on surfaces in these facilities, including walls, window edges, flooring, benches, and shared items like slippers [50,51]. These findings highlight the importance of personal hygiene practices and routine disinfection in minimizing fungal transmission in such communal environments.

Although this study noted a potential association between contact with farm animals and an increased risk of dermatophytosis, the relationship was not statistically significant. Nevertheless, prior research from Poland [13] and Romania [52] has documented the zoonotic transmission of dermatophytes through direct contact with infected livestock or contaminated elements in their habitats. Poor hygiene can amplify this transmission risk, underscoring the need for targeted prevention strategies in agricultural settings.

In our study, schoolchildren exhibited higher rates of dermatophyte infection compared to other demographic groups, although the difference was not statistically significant. Studies from Nigeria [53] and Indonesia [54] similarly reported elevated infection rates among school-aged children. These findings are likely attributable to increased physical activity, frequent participation in group activities, and lower adherence to personal hygiene practices, which collectively create favorable conditions for fungal infections in this population.

This study is limited by the absence of data on antifungal resistance, which restricts a comprehensive understanding of treatment outcomes. Additionally, the cross-sectional design and sampling methods preclude the assessment of temporal relationships between variables, limiting the generalizability of the findings to the broader population.

## 5. Conclusions

The present study demonstrated that dermatophytosis was identified in nearly one third of the patients examined, with *Microsporum* spp. emerging as the predominant causative agent. The most frequently observed clinical manifestation was Tinea corporis, followed by Tinea capitis. Certain demographic and behavioral factors were associated with a higher or significantly higher risk of infection, including male gender, age under 10 years, rural residence, a family history of dermatophytosis, contact with cats, sexual contact with an infected or unknown partner, and frequent visits to bathhouses, massage parlors, or sports facilities.

These findings underscore the need to enhance preventive measures and diagnostic strategies for dermatophytosis, particularly among high-risk groups. Healthcare practitioners should remain vigilant about additional risk factors, such as a history of contact with infected animals or family members, and exposure to communal environments like public baths, sports facilities, and massage parlors. Early detection and intervention in these contexts could significantly reduce the burden and spread of the disease.

Future research should focus on assessing dermatophyte resistance to leading antifungal agents using advanced laboratory techniques. Additionally, the data presented in this study highlight new avenues for scientific inquiry into the diagnostics and treatment of dermatophytosis, potentially paving the way for the development of more effective prevention and therapeutic strategies.

## Figures and Tables

**Table 1:** Socio-demographic characteristics of patients with suspected dermatophytosis treated at the dermatology unit of City Multidisciplinary Hospital No. 3 in Astana, Kazakhstan (n = 313).

Demographic Characteristics		N	(%)
Gender	Male	175	55.9
	Female	138	44.1
Age (years)	1–10	78	24.9
	11–20	80	25.6
	21–30	82	26.2
	>30	73	23.3
Place of residence	Countryside	57	18.2
	City	256	81.8
Occupation	Kindergarten visitor	42	13.4
	Schoolchild	104	33.2
	Student	36	11.5
	Housekeeper	36	11.5
	Office worker	63	20.1
	Others	32	10.2

**Table 2:** Clinical characteristics of patients with suspected and confirmed dermatophytosis treated at the dermatology unit of City Multidisciplinary Hospital No. 3 in Astana, Kazakhstan (n = 313).

Characteristics		Frequency	%
Contact with a sick relative	Yes	42	13.4
	No	271	86.6
Contact with a cat	Yes	94	30.0
	No	219	70.0
Sexual contact with an infected or unknown partner	Yes	48	15.3
	No	265	84.7
Contact with farm animals	Yes	45	14.4
	No	268	85.6
Visiting a massage parlor	Yes	34	10.9
	No	279	89.1
Visiting a sports section	Yes	59	18.8
	No	254	81.2
Visiting a bathhouse	Yes	56	17.9
	No	257	82.1

**Table 3:** Clinical and mycological profile among patients with suspected and confirmed dermatophytosis.

Characteristic		Microscopy		Culture		Clinical Manifestations		
		Positive	Negative	Positive	Negative	Tinea Capitis	Tinea Corporis	Tinea Cruris
Gender	Male	133	42	67	108	47	60	26
	Female	79	59	28	110	17	53	9
Age	1–10	66	12	34	44	30	36	0
	11–20	56	24	23	57	23	30	3
	21–30	55	27	24	58	7	28	20
	>30	35	38	14	59	4	19	12
Place of residence	City	167	89	76	180	50	88	29
	Village	45	12	19	38	14	25	6
Occupation	Kindergarten visitor	31	11	15	27	20	11	0
	Schoolchild	84	20	42	62	35	48	1
	Student	26	10	11	25	5	15	6
	Housekeeping	19	17	9	27	1	10	8
	Office worker	36	27	12	51	1	19	16
	Others	16	16	6	26	2	10	4
Contact with a sick relative	Yes	32	10	15	27	14	17	1
	No	180	91	80	191	50	96	34
Contact with a cat	Yes	70	24	37	57	38	32	0
	No	142	77	58	161	26	81	35
Sexual contact	Yes	36	12	12	36	3	6	27
	No	176	89	83	182	61	107	8
Contact with farm animals	Yes	34	11	16	29	10	19	5
	No	178	90	79	189	54	94	30
Visiting a massage parlor	Yes	26	8	10	24	2	15	9
	No	186	93	85	194	62	98	26
Visiting a sports section	Yes	48	11	15	44	9	36	3
	No	164	90	80	174	55	77	32
Visiting a bathhouse	Yes	42	14	21	35	7	32	3
	No	170	87	74	183	57	81	32

**Table 4:** Bivariate and multivariate logistic regression analyses of social and epidemiological factors of dermatophytosis, 2023.

Variables	Categories	Dermatophytosis		COR 95% CI	*p*-Value	AOR 95% CI	*p*-Value
		+ (%)	– (%)				
Gender	Male	133 (76.0)	42 (24.0)	2.37 (1.46–3.84)	<0.001	1.97 (1.14–3.39)	0.015
	Female	79 (57.2)	59 (42.8)	1	-	1	-
Age	1–10	66 (84.6)	12(15.4)	5.97 (2.77–12.87)	<0.001	3.80 (1.01–4.31)	0.048
	11–20	56 (70.0)	24 (30.0)	2.53 (1.31–4.92)	0.006	1.09 (0.33–3.62)	0.891
	21–30	55 (67.1)	27 (32.9)	2.21 (1.15–4.24)	0.017	2.03 (0.86–4.81)	0.109
	>30	35 (47.9)	38 (52.1)	1	-	1	-
Place of residence	City	167 (65.2)	89 (34.8)	1	-	1	-
	Village	45 (78.9)	12 (21.1)	2.00 (1.01–3.97)	0.048	2.30 (1.04–5.09)	0.040
Occupation	Kindergarten visitor	31 (73.8)	11 (26.2)	2.82 (1.06–7.48)	0.038	1.36 (0.29–6.34)	0.698
	Schoolchild	84 (80.8)	20 (19.2)	4.20 (1.80–9.80)	0.001	2.20 (0.58–8.36)	0.247
	Student	26 (72.2)	10 (27.8)	2.60 (0.95–7.11)	0.063	2.09 (0.56–7.83)	0.272
	Housekeeping	19 (52.8)	17 (47.2)	1.12 (0.43–2.90)	0.819	1.17 (0.39–3.55)	0.783
	Office worker	36 (57.1)	27 (42.9)	1.33 (0.57–3.13)	0.509	1.05 (0.36–3.06)	0.925
	Others	16 (50.0)	16 (50.0)	1	-	1	-
Contact with a sick relative	Yes	32 (76.2)	10 (23.8)	1.62 (0.76–3.44)	0.211	3.04 (1.22–7.55)	0.017
	No	180 (66.4)	91 (33.6)	1	-	1	-
Contact with a cat	Yes	70 (74.5)	24 (25.5)	1.58 (0.92–2.72)	0.096	2.32 (1.12–4.81)	0.024
	No	142 (64.8)	77 (35.2)	1	-	1	-
Sexual contact	Yes	36 (75.0)	12 (25.0)	1.52 (0.75–3.06)	0.244	3.08 (1.34–7.10)	0.008
	No	176 (66.4)	89 (33.6)	1	-	1	-
Contact with farm animals	Yes	34 (75.6)	11 (24.4)	1.56 (0.76–3.23)	0.228	1.09 (0.46–2.58)	0.852
	No	178 (66.4)	90 (33.6)	1	-	1	-
Visiting a massage parlor	Yes	26 (76.5)	8 (23.5)	1.63 (0.71–3.73)	0.252	1.39 (1.23–8.28)	0.017
	No	186 (66.7)	93 (33.3)	1	-	1	-
Visiting a sports section	Yes	48 (81.4)	11 (18.6)	2.40 (1.19–4.84)	0.015	3.21 (1.26–8.14)	0.014
	No	164 (64.6)	90 (35.4)	1	-	1	-
Visiting a bathhouse	Yes	42 (75.0)	14 (25.0)	1.54 (0.80–2.96)	0.201	2.32 (1.02–5.28)	0.045
	No	170 (66.1)	87 (33.9)	1	-	1	-

## Data Availability

The original contributions presented in this study are included in the article; further inquiries can be directed to the corresponding author.
